# Hairy Cell Leukemia: Hematological and Immunophenotypic Profile of 13 Patients

**DOI:** 10.7759/cureus.44876

**Published:** 2023-09-07

**Authors:** Vandana Bhatti, Gurpreet Kaur, Aarzoo Ahuja, Roma Isaacs

**Affiliations:** 1 Department of Pathology, Christian Medical College & Hospital, Ludhiana, IND

**Keywords:** immunophenotyping, hcl-v, hcl, hairy cell leukemia, b-cell lymphoproliferative disorder

## Abstract

Introduction: Hairy cell leukemia (HCL) is a rare lymphoproliferative disorder of the mature B-cells, mostly seen in men, and is characterized by cytopenia, splenomegaly, myelofibrosis, and the presence of atypical lymphoid cells showing the cytoplasmic hairy projection in the peripheral blood, bone marrow, and spleen. The immunophenotypic (IPT) profile shows the clonal expansion of B-cells with CD19, CD20, and CD22 showing bright expression. The diagnosis requires two hairy cell markers out of CD103, CD123, CD25, and CD11c to be positive. The HCL variant (HCL-v) has a different IPT profile with negative CD25 in most cases.

Aim: The aim was to study the hematological and IPT of classical HCL and HCL variants.

Methods: This cross-sectional study included all the cases of HCL diagnosed over a retrospective period of eight years from 1st January 2015 to 31st December 2022 in a tertiary care hospital in north India. The patients included in the study were those for whom immunophenotyping; that is, flow cytometry and/or immunohistochemistry (IHC) were done for diagnosis. Bone marrow slides, IHC slides, and flow cytometric IPTs were reviewed.

Results: The study included 13 patients who were diagnosed to have HCL, of which 12 were classical HCL and one was HCL-variant (HCL-v). Among classical HCL, IPT was done by flow cytometry in 10 patients, while in two patients, it was done by IHC. CD19, CD20, and CD22 were positive in all patients of classical HCL (10/10, 10/10, and 5/5, respectively), while CD123, CD103, CD25, and CD11C were positive in 100%, 89%, 80%, and 100% cases, respectively. One patient of HCL-v had CD103 and CD123 positive, while CD25 and CD123 were negative.

Conclusion: The diagnosis of HCL requires a multipronged approach. The use of clinical features, morphology, and immunophenotyping combined with ancillary techniques provides higher diagnostic accuracy and enables its distinction from other B-cell lymphoproliferative disorders (BCLPDs), leading to better patient management and treatment.

## Introduction

Hairy cells are transformed B-cells originating in the marginal zone of the lymph nodes and spleen [1]. Hairy cell leukemia (HCL) is a lymphoproliferative disorder of the mature B-cells, which is characterized by cytopenia, splenomegaly, myelofibrosis, and the presence of atypical lymphoid cells showing cytoplasmic hairy projection in the peripheral blood, bone marrow, and spleen [2]. It is a rare disease accounting for 2% of leukemias and occurs more frequently in men as compared to women [1,3]. HCL is a disease of middle-aged to elderly adults with an average of 58 years, uncommon in the teenage or pediatric population [1]. Occupations that involve exposure to diesel fuel, organic solvents, large animal farming, and pesticide and herbicide exposure are involved in the development of the disease; however, the epidemiology is still only partially understood [4]. A specific morphological feature is the accumulation of monoclonal B-cells showing hair-like circumferential cytoplasmic projections on its surface, which are seen in peripheral blood and bone marrow [5].

Sometimes morphological features can mislead the diagnosis to splenic marginal zone lymphoma, prolymphocytic leukemia, HCL-v, and mantle cell lymphoma [5]. The distinction of HCL from these is important for its timely management [6]. The morphological assessment can sometimes be difficult due to dry tap aspiration, because of marked reactive fibrosis, which is a common presentation [7,8].

HCL has a characteristic immunophenotypic (IPT) profile, which helps in its differentiation from other mature B-cell chronic lymphoproliferative neoplasms [9]. The characteristic IPT shows the clonal expansion of B-cells with CD19, CD20, and CD22 showing bright expression, and positivity for CD103, CD11C, CD25, FMC7, and SmIg ++, while CD5 and D23 are most of the time negative [2,3].

BRAF mutation has been linked to HCL and is present in 85% of the cases [3]. It is absent in HCL-variant (HCL-v). The absence of BRAF mutation and annexin-A1 in HCL-v suggests that HCL and HCL-v are two different entities [10]. Thus, a holistic approach is required for diagnosing HCL, including clinical findings, morphology, Immunophenotyping, and other ancillary techniques.

The present study provides the hematological and IPT of HCLs and the importance of the holistic approach in its diagnosis.

## Materials and methods

This eight-year cross-sectional retrospective study included all the cases diagnosed as HCL (classical and variant) on bone marrow and immunophenotyping (flow cytometry or/and Immunohistochemistry (IHC)) over a period of eight years from 1st January 2015 to 31st December 2022 in Christian Medical College and Hospital, Ludhiana, Punjab, India. The cases of HCL in which immunophenotyping was done by flow cytometry and/or IHC were included in the study. HCL cases in which immunophenotyping was not done were excluded from the study. Peripheral blood smears, May Grunwald-Giemsa stained bone marrow aspirate slides, hematoxylin and eosin stained trephine biopsies, IHC slides, and flow cytometric immunophenotyping reports of enrolled patients were retrieved from the records and reviewed. The relevant clinical information, which included presenting symptoms and organomegaly, was obtained from medical records. Data were entered using Microsoft Excel and analyzed using percentages and proportions. Descriptive analysis was done with measures of variants including mean, median, and standard deviation for continuous variables. Nominal variables were expressed as percentages. The study was approved by the institutional research (CMC/2272) and ethics committee (BMHR-IEC/Approval-R-Proj/23-08-287/Patho). Consent for bone marrow aspiration/biopsy was taken prior to the procedure.

## Results

There were 13 patients who were diagnosed to have HCL. The mean age was 58.3±11.4 years with male predominance (M:F was 3:1). All 13 patients had splenomegaly at the time of diagnosis. Out of 13 patients with HCL, 12 were classical HCL, and one was HCL-v.

Classical HCL

Among the classical HCL patients, splenomegaly was the most common clinical feature in all 12/12(100%) patients, followed by hepatomegaly in 9/12 (75%) patients. The other clinical features seen in classical HCL patients are shown in Table 1.

**Table 1:** Clinical features in classical hairy cell leukemia

Clinical features	N=12	Percentage (%)
Splenomegaly	12	100
Hepatomegaly	9	75
Fever	6	50
Weakness	6	50
Pallor	6	50
Bleeding	4	34
Weight loss	3	25
Abdominal pain	2	17
Lymphadenopathy	2	17

**Figure 1:**
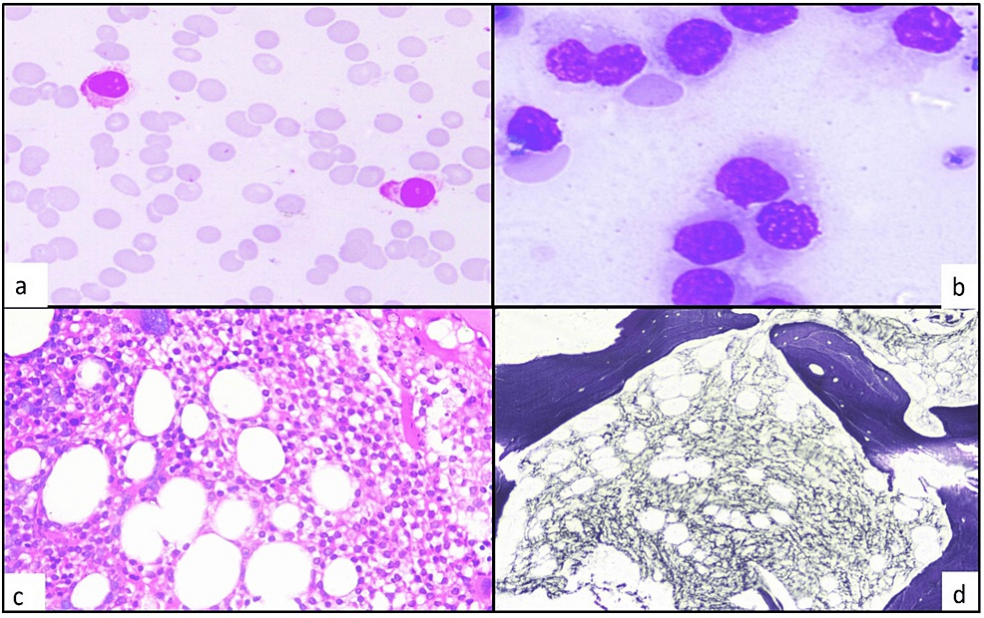
a-d: Classical hairy cell leukemia. a) Peripheral blood smear shows hairy cells with fine hairy projections (400x, Leishman stain). b) Bone marrow aspirate shows many hairy cells with fragile cytoplasm (1000x, May Grunwald-Giemsa stain). c) Trephine biopsy shows fried egg appearance (400x, Hematoxylin and eosin stain). d) Reticulin stain shows increased fibrosis (400x, Gordon and Sweet’s Silver stain)

Pancytopenia was observed in 58.33% (7/12) of patients, while 41.66% (5/12) had bicytopenia. Other hematological parameters showed median hemoglobin, TLC, and platelet count of 8.6 g/dl, 2,900/cu.mm, and 49,000/cu.mm, respectively. Hairy cells were seen in peripheral blood smear (PBS) in 66.6% (8/12) patients. Bone marrow aspirate (BMA) was a dry tap in 25% (3/12) patients. Bone marrow trephine biopsy was hypercellular in 92% (11/12), while it was hypocellular in 8% (1/12). Trephine biopsy showed infiltration by lymphoid cells in all (12/12), while 17% (2/12) showed a characteristic fried egg appearance (Figure 1).

**Figure 2:**
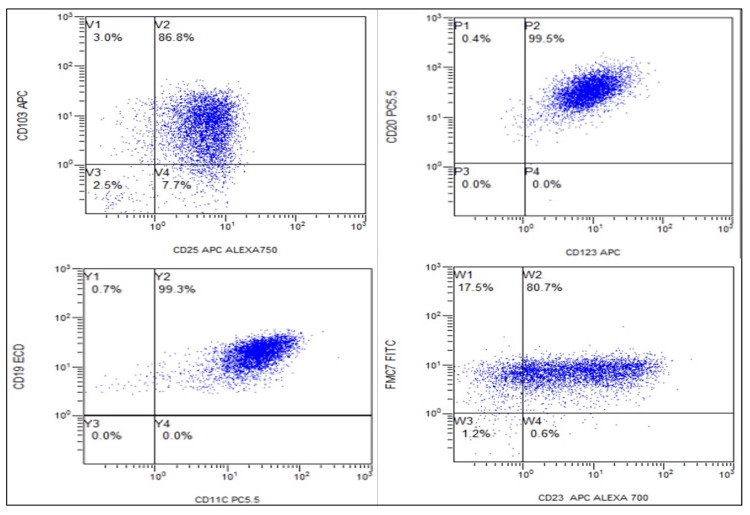
Flowcytometric dot plots in classical hairy cell leukemia showing positivity for CD103, CD25, CD20, CD123, CD19, CD11C, FMC 7, and CD23

Bone marrow fibrosis was 4+ in one (8.33%), 3+ in seven patients (58.33%), 2+ in three (25.00%), and 1+ in one (8.33%). Immunophenotyping was done by flow cytometry in 10 patients, while in two patients, it was done by IHC. Flow cytometric immunophenotyping revealed that CD5 was negative in all nine cases in which it was done (0/9) and CD10 was negative in all six cases in which it was done (0/6). The B-cell-specific markers, CD19, CD20, and CD22, were positive in all patients (10/10, 10/10, and 5/5, respectively). Hairy cell-specific markers comprising CD123, CD103, CD25, and CD11C were positive in 100%, 89%, 80%, and 100% cases, respectively (Figure 2).

IHC was done on two patients, showing CD20 and annexin A1 positivity. CD5 was negative in both; however, CD10 was positive in one patient. CD23 was done in one patient, which was negative. BRAF V600E mutation was done in seven patients and was positive in 6/7 (86%) patients.

**Figure 3:**
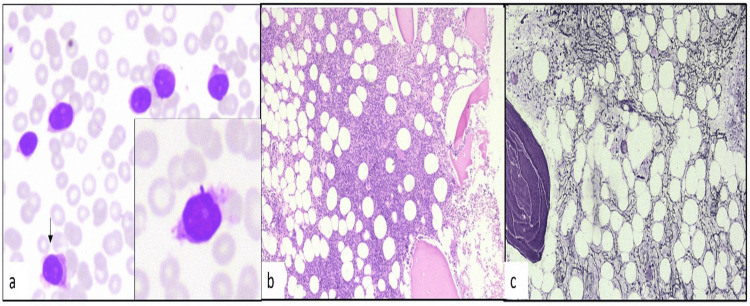
a-c: Hairy cell leukemia variant. a) Peripheral blood smear shows many lymphoid cells with an arrow showing a hairy cell. The inset shows hairy cells with fragile cytoplasm and prominent nucleoli (400x, Leishman stain).b) Trephine biopsy shows lymphoid cells (100x, Hematoxylin and eosin stain).c) Reticulin stain shows increased fibrosis. (400x, Gordon and Sweet’s Silver stain)

HCL-v: There was one case of HCL-v. This was a 71-year-old gentleman who presented with fever, weakness, weight loss, and abdominal fullness. On examination, he had pallor and splenomegaly. His complete blood count showed bicytopenia with Hb of 9.9 gm/dl and platelet count of 71,000/cu.mm. He had leukocytosis (TLC=86,800/cu.mm), and peripheral blood smear showed an increase in atypical lymphoid cells having a high N:C ratio, round nucleus with conspicuous nucleoli, and a moderate amount of cytoplasm with circumferential hairy cytoplasmic projections (Figure 3). Monocytopenia was absent.

BMA was hypercellular, and trephine biopsy showed interstitial involvement by a small to intermediate-sized lymphoid cell having a high N:C ratio, coarse chromatin, and small to moderate cytoplasm. Flow cytometric immunophenotyping showed CD5 and CD10 negativity with positive B-cell markers comprising CD19, CD20, and CD22, along with positive CD 103, CD11c, FMC-7, and CD23. However, CD25 and CD123 were negative.

**Figure 4:**
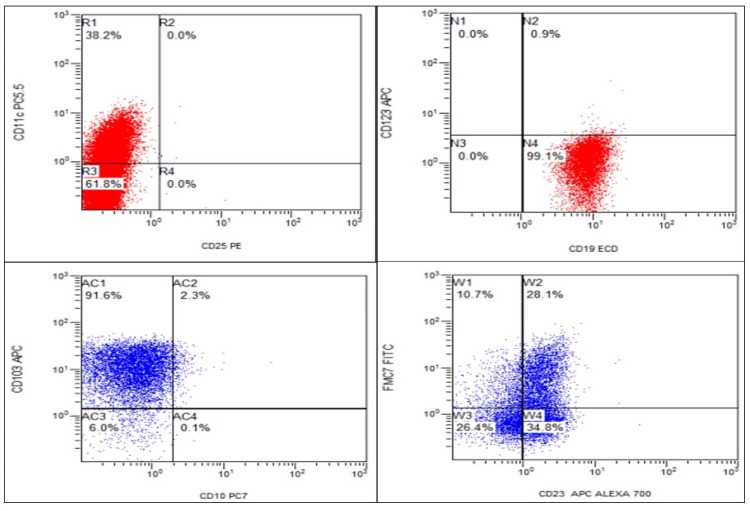
Flowcytometric dot plots in the hairy cell leukemia variant show positivity for CD11c, CD103, CD 19, FMC 7, and CD23. CD25, CD123, and CD10 are negative

Annexin A1 IHC done on trephine biopsy was negative, and BRAF V600E mutation was not detected (Figure 4).

## Discussion

The diagnosis of HCL is based on morphology, i.e., the presence of hairy cells in the peripheral blood and bone marrow, and associated with pancytopenia and splenomegaly, eventually resulting in myelofibrosis [3]. Immunophenotyping and mutation studies aid in confirming the diagnosis along with other findings. In the present study, the mean age of patients was 58 years, and there was a male preponderance with M:E ratio of 3:1. Similar findings were observed by Gaman with a mean age of 52 years and M:E ratio of 3:1 [2].

The most common clinical feature in our study was splenomegaly, which was observed in all cases (100%), followed by hepatomegaly in 84% of cases. Gaman in her study observed splenomegaly in 70% of cases. Hematological parameters showed median hemoglobin, TLC, and platelet count of 8.6 g/dl, 2,900/cu.mm, and 49,000/cu.mm, respectively, in our study [2]. Gaman in her study also reported similar findings with median hemoglobin of 10.3g/dl, TLC of 2,400/cu.mm, and platelet count of 83,000/cu.mm [2]. Pancytopenia in classical hairy cell leukemia was present in 58.3% (7/12) cases, while it was observed in 61.5% of cases by Gaman [2]. Hairy cells were present in 66.6% (8/12) cases of classical HCL, while Angelova et al. observed hairy cells in peripheral blood smears in 100% of patients (22/22) [11].

In our study, BMA was a dry tap in 25% (3/12) cases, while Galani et al. in their study observed bone marrow dry tap in 84% (21/25) cases [9].

We observed that the characteristic fried egg appearance in trephine biopsy was present in 2/12 (17%) and bone marrow fibrosis was grade 4+ in one patient (8.3%), 3+ in seven (58.3%), 2+ in three (25.0%), and 1+ in one patient (8.3%), while Bhargava et al. found fried egg appearance in 12/18(66.7%) patients and reticulin fibrosis of grades 2-3 [12].

Flow cytometric immunophenotyping was performed in 10 out of 12 patients and is shown in Table 2.

**Table 2:** Flowcytometric immunophenotypic profile of classical hairy cell leukemia

Markers	N=10 (%)	N=68 (100%), Maitre et al. [11]	N=169 (100%), Shao et al. [10]
CD19	10 (100%)	68/68 (100%)	168/169 (99%)
CD20	10 (100%)	68/68 (100%)	168/169 (99%)
CD22	5/5 (100%)	-	169/169 (100%)
CD25	8/10 (80%)	68/68 (100%)	169/169 (100%)
CD103	8/9 (89%)	66/67 (99%)	169/169 (100%)
CD123	8/8 (100%)	60/62 (97%)	114/114 (100%)
CD11c	10/10 (100%)	68/68 (100%)	167/169 (99%)
FMC7	7/9 (78%)	-	-
CD23	8/10 (80%)	22/68 (32%)	36/169 (21%)
CD5	0/9 (0%)	7/68 (10%)	4/169 (2%)
CD10	0/6 (0%)	12/68 (18%)	21/169 (12%)

CD5 was negative in all nine cases in which it was done (0/9) and CD10 was negative in all six cases in which it was done (0/6). CD 5 and CD 10 negative phenotype is seen in cases of hairy cell leukemia but is not specific to it. It helps in narrowing down the list of differential diagnoses [13]. The B-cell-specific markers, CD19, CD20, and CD22, were positive in all patients (10/10, 10/10, and 5/5, respectively). Hairy cell-specific markers comprising CD123, CD103, CD25, and CD11C were positive in 100%, 89%, 80%, and 100% cases, respectively. The comparison of these markers with other studies is depicted in Table 2. CD25 was positive in 80% (8/10) of cases, whereas other studies showed positivity in 100% of cases [10,14]. However, the two cases that were negative for CD25 were positive for BRAF V600E mutation and hence were labeled as classical HCL. Because of financial constraints, BRAF V600E mutation was done in only seven cases, and it was positive in 86% (6/7), while Shao et al. found BRAF V600E positivity in 76% of cases [10].

Annexin IHC was done on two patients of classical HCL and was positive in both (100%). Sherman et al. in their study found annexin positivity in 96% of patients with classical HCL (95/99) [15].

HCL-v: In our study, there was one case of HCL-v. The patients of HCL-v have high TLC, lack monocytopenia, and have hairy cells with prominent nucleoli in peripheral blood smear which is not seen in classical HCL [6]. Our patient also had all these findings.

The immunophenotyping of HCL-V in our patient showed CD5- and CD10- patterns with the presence of B-cell-specific markers, i.e., CD19/CD20/CD22. CD103 and FMC7 were positive, which is in concordance with a study done by Angelova et al. and Maitre et al. [11,14]. CD 11c was also positive, while CD25 and CD123 were negative (Table 3).

**Table 3:** Flowcytometric immunophenotypic profile of the hairy cell leukemia variant

	N=1 Present study	Angelova et al. [14]	Shao et al. [10]
CD19	1/1	22/22 (100%)	35/35 (100%)
CD20	1/1	21/21 (100%)	35/35 (100%)
CD22	1/1	18/18 (100%)	35/35 (100%)
FMC7	1/1	10/11 (91%)	-
CD23	1/1	0/16 (0%)	5/35 (14%)
CD11c	1/1	21/21 (100%)	35/35 (100%)
CD25	0/1	3/22 (14% )	0/35 (0%)
CD103	1/1	21/22 (95%)	35/35 (100%)
CD123	1/1	1/1 (100% )	8/20 (40%)
CD 5	0/1	0/1 (0%)	1/35 (3%)
CD 10	0/1	0/1 (0%)	5/35 (14%)

Shao et al. found CD 25 negativity in all patients (0/35). However, CD123 was positive in 40% of patients (8/20) [10]. BRAF V600E was not detected.

Our study had the limitation of a small sample size as HCL is a rare disease. Thus, the diversity of disease within the broader population may not be fully captured. Further studies involving a larger population would allow a better understanding of the hematological and immunophenotypic profile of this disease.

## Conclusions

HCL has a distinct morphological and immunophenotypic profile. HCL-v differs from classical HCL haematologically and immunophenotypically. This rare chronic lymphoproliferative disorder requires a combined approach for its definitive diagnosis, which includes clinical features, morphology, immunophenotyping, and mutation studies. A combined approach facilitated early detection and precise classification of HCL in our study leading to early and proper initiation of therapy.
